# Identification of Apple Leaf Diseases by Improved Deep Convolutional Neural Networks With an Attention Mechanism

**DOI:** 10.3389/fpls.2021.723294

**Published:** 2021-09-28

**Authors:** Peng Wang, Tong Niu, Yanru Mao, Zhao Zhang, Bin Liu, Dongjian He

**Affiliations:** ^1^College of Mechanical and Electronic Engineering, Northwest A&F University, Xianyang, China; ^2^Key Laboratory of Agricultural Internet of Things, Ministry of Agriculture and Rural Affairs, Xianyang, China; ^3^Shaanxi Key Laboratory of Agricultural Information Perception and Intelligent Services, Xianyang, China; ^4^College of Information Engineering, Northwest A&F University, Xianyang, China

**Keywords:** apple disease, CA-ENet, attention mechanism, CA block, diseases identification

## Abstract

The accurate identification of apple leaf diseases is of great significance for controlling the spread of diseases and ensuring the healthy and stable development of the apple industry. In order to improve detection accuracy and efficiency, a deep learning model, which is called the Coordination Attention EfficientNet (CA-ENet), is proposed to identify different apple diseases. First, a coordinate attention block is integrated into the EfficientNet-B4 network, which embedded the spatial location information of the feature by channel attention to ensure that the model can learn both the channel and spatial location information of important features. Then, a depth-wise separable convolution is applied to the convolution module to reduce the number of parameters, and the h-swish activation function is introduced to achieve the fast and easy to quantify the process. Afterward, 5,170 images are collected in the field environment at the apple planting base of the Northwest A&F University, while 3,000 images are acquired from the PlantVillage public data set. Also, image augmentation techniques are used to generate an Apple Leaf Disease Identification Data set (ALDID), which contains 81,700 images. The experimental results show that the accuracy of the CA-ENet is 98.92% on the ALDID, and the average F1-score reaches .988, which is better than those of common models such as the ResNet-152, DenseNet-264, and ResNeXt-101. The generated test dataset is used to test the anti-interference ability of the model. The results show that the proposed method can achieve competitive performance on the apple disease identification task.

## Introduction

The apple industry is one of the most important fruit industries in China. However, the frequent occurrence of apple leaf diseases may seriously restrict the healthy and stable development of the apple industry. At present, the diseases of a large number of industrialized apple orchards mainly rely on human vision for recognition, which requires a high degree of reliance on disease experts. The identification task is huge, especially since the visual inspection of fruit farmers or experts is prone to misjudgment due to their subjective perception and visual fatigue, and it is difficult to meet the demand for high-precision identification for intelligent orchards (Dutot et al., 2013). The problems previously discussed will lead to a large lag in the tracking management process of orchard diseases, which causes the improper use of pesticides and reduces the quality of fruit. Therefore, the accurate identification of diseases is of great significance to improve the yield and quality of apples and to cultivate disease-resistant varieties.

With the development of computer vision, machine learning techniques have been widely used in the agricultural field in recent years, and a series of approaches have been achieved in crop disease identification (Aravind et al., 2018; Kour and Arora, 2019; Mohammadpoor et al., 2020). In recent years, the main techniques, which are widely used in crop disease identification include artificial neural network (ANN) (Sheikhan et al., 2012), the K Nearest Neighbors (KNN) algorithm (Guettari et al., 2016), random forests (RF) (Kodovsky et al., 2012), and so on. For example, Wang et al. (2019) proposed a method for identifying cucumber powdery mildew based on a visible spectrum by extracting the spectral features and training a Support Vector Machine (SVM) classifier to establish a classification model, optimizing the radial basis kernel function, and the recognition accuracy of the method reached 98.13%. In contrast, Prasad et al. (2016) proposed a mobile client-server architecture for leaf disease detection and diagnosis based on the combination of a Gabor Wavelet Transform (GWT) and a Gray-Level Co-occurrence Matrix (GLCM). The mobile terminal captures the object image and then transmits it to the server after pre-processing. The server then performs GWT-GLCM feature extraction and classification based on the KNN algorithm. The system can monitor farmland information through the mobile terminal at any stage. Although the previously discussed studies achieved outstanding performances in disease identification tasks, the low-level feature representations extracted from them are limited to intuitive shallow features, such as the colors, textures, and shapes of the images. Thus, it is difficult to achieve competitive performance on apple leaf disease identification tasks.

Compared with machine learning algorithms that require cumbersome image pre-processing and feature extraction (Kulin et al., 2018; Zhang et al., 2018b), convolutional neural networks (CNNs) can directly learn robust high-level feature representations of apple diseases from images. The extracted high-level feature representation is richer and better compared with the method of manually extracting features; therefore, CNNs have achieved excellent results in multiple visual tasks (Ren et al., 2017; Liu et al., 2018; Bi et al., 2020). In recent years, with the continuous emergence of advanced deep learning architectures such as the ResNet (He et al., 2016), ResNeXt (Xie et al., 2017), and DenseNet (Huang et al., 2017), the recognition accuracy and speed are constantly being refreshed on the public dataset, ImageNet. In order to solve the problem of the mobile deployment of the model, scholars have proposed various lightweight architectures, such as Xception (Chollet, 2017), MobileNet (Howard et al., 2017; Sandler et al., 2018), ShuffleNet (Ma et al., 2018; Zhang et al., 2018a), and so on. In order to provide a stable, efficient, low-cost, and highly intelligent disease identification method, Chao et al. (2020) proposed that the XDNet combined with DenseNet and Xception can enhance the feature extraction capability of the model. The model achieved an accuracy of 98.82% in identifying five apple leaf diseases with fewer parameters. Liu et al. (2020) adopted the Inception structure and introduced a dense connection strategy to build a new neural network model, which realized the real-time and accurate identification of six different kinds of grape leaf diseases. In addition, Ramcharan et al. (2019) deployed a trained cassava disease recognition model for a mobile terminal. Tests under natural conditions in the field found that complex conditions, such as different angles, brightness, and the occlusion of the image taken, could adversely affect the performance of the model, which also proves that image classification under the complex background of the field is challenging.

An attention mechanism can provide a novel solution for feature extraction. The attention mechanism can assign larger weights to regions of interest and smaller weights to backgrounds and extract information that contributes more to classification to optimize the model and to make judgments that are more accurate. In other studies, attention mechanisms have achieved excellent performance in tasks, such as classification, detection, and segmentation (Hu et al., 2018; Karthik et al., 2020; Mi et al., 2020; Hou et al., 2021). Inspired by the above researches, this study proposes a new CNN for apple diseases recognition. The main contributions and innovations of this study are summarized as follows:

1. A new Apple Leaf Disease Identification Data set (ALDID) is generated by using image generation techniques. In order to enhance the generalization performance of the model, image augmentation techniques are used to expand the data set and simulate apple leaf disease images collected under different conditions, laying a foundation for the training of the model.
2. A novel attention-based apple leaf disease recognition model, namely, the Coordination Attention EfficientNet (CA-ENet), is proposed. A network search technique is first used to determine the optimal structure of the model, and the optimal parameters of network depth, width, and input image resolution are obtained. Then, the deep separable convolution is applied to the coordination attention convolution (CA-Conv) infrastructure to greatly reduce the number of parameters and avoid an overfitting problem. Finally, a coordinated attention block is embedded in the infrastructure to realize the integration of characteristic channel information and spatial information attention and to strengthen the learning ability of the model for important information in the lesion area.

The remainder of the study is organized as follows: In section Materials and Methods, the detailed information of the dataset is introduced and expanded by data augmentation techniques. The model proposed in this study and the related content of attention visualization is introduced in detail. The section Results and Discussion presents the experiments for evaluating the performance of the model and analyzes the results of the experiments, discussed the impact of data augmentation and external interference on the performance of the model. The last section, Conclusion and Future Work, summarizes the work of this study and prospects for further research.

## Materials and Methods

This section introduces the materials and methods used in the study in detail, including the collected apple diseased leaf images and the ALDID established after augmentation. It also presents the proposed model and the attention visualization method.

### Image Acquisition

The study was conducted from July 2020 to October 2020, at the apple planting experimental station of the Northwest A&F University in Qianxian County, Shaanxi province. By using a variety of different types of mobile devices, a huge number of field environment apple leaf images under different angles and distances are collected. There are a total of 5,170 disease images with a resolution of 3,000 × 3,000 pixels, including those of five species of the Glomerella leaf spot (*Colletotrichum fructicola*), Apple leaf mites (*Panonychus ulmi*), Mosaic (Apple mosaic virus), Apple litura moth (*Spodoptera litura Fabricius*), and Healthy leaves. In addition, 3,000 disease images under a single background of three kinds of laboratories, namely, Black rot (*Physalospora obtuse*), Scab (*Venturia inaequalis*), and Rust (*Gymnosporangium yamadai*), were collected from the public dataset PlantVillage. The above two data sets are shuffled and mixed to generate the original data set of common apple diseases.

**Figure 1:**
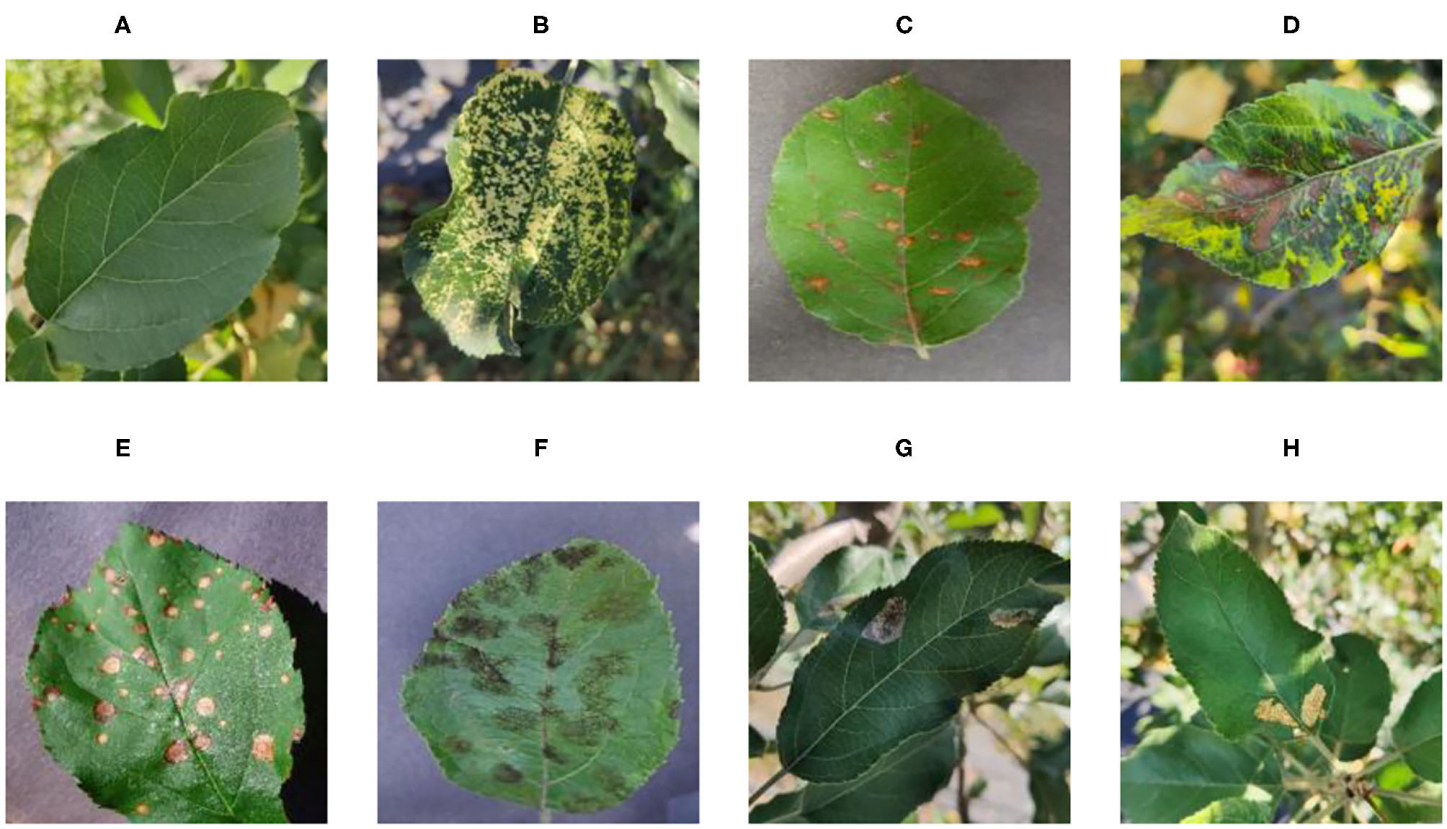
Eight common apple leaf disease types. **(A)** Healthy, **(B)** Mosaic, **(C)** Rust, **(D)** Glomerella leaf spot, **(E)** Black rot, **(F)** Scab, **(G)** Apple litura moth, and **(H)** Apple leaf mites.

Figure 1 shows random samples of each category in the data set. There are a large number of complex background images in the data set. At the same time, it can be seen that Apple litura moth (G) and Apple leaf mites (H) leaves have relatively similar geometric features. The difference between the two diseases can be expressed as a fine-grained image classification problem. A variety of different forms of samples can increase the diversity of the data set, making it closer to various different situations that may occur in the real situation. However, it also constitutes a greater test for the image classification task and puts forward higher requirements for the comprehensive performance of the model.

### Image Augmentation

When acquiring the apple disease images, the samples obtained varied in the apple leaf growth position, weather condition, shooting angle, and there are interference factors such as equipment noise. In order to enable the model to learn as many irrelevant patterns as possible and avoid overfitting problems, the images of the dataset need to be expanded and normalized.

**Figure 2:**
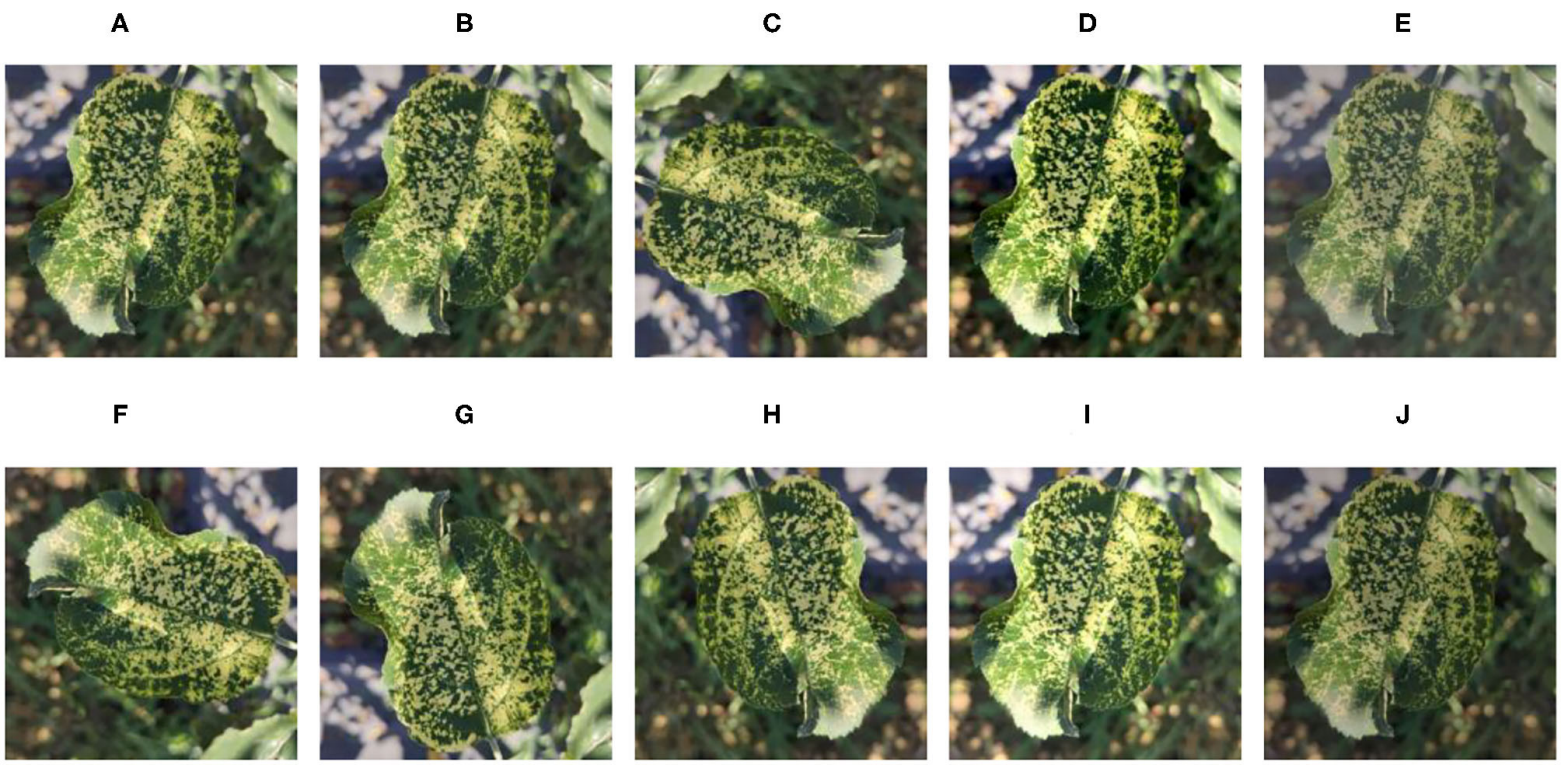
Image enhancement example of the mosaic disease. **(A)** Original image, **(B)** Gaussian blur, **(C)** 90° rotation, **(D)** High contrast, **(E)** Low contrast, **(F)** 270° rotation, **(G)** Horizontal symmetry, **(H)** Vertical symmetry, **(I)** High brightness, and **(J)** Low brightness.

In the data expansion, Gaussian blurring, contrast enhancement by 30% and decrease by 30%, and brightness enhancement by 30% and decrease by 30% are adopted to simulate different weather conditions for all samples of the original dataset. The images are also rotated by 90°, 270°, a horizontal flip, and a vertical flip to simulate the change of shooting angle, then the original data set is added. A Mosaic disease image is randomly selected to enhance and display the effect as shown in Figure 2. Table 1 represents the structure information of the ALDID. It can be seen from Table 1 that the sample distribution is balanced after image expansion, which is in line with the actual application scenario. It can ensure that the model extracts different features of each category in a balanced manner, ensuring its correct training and avoiding overfitting. This study also divides the ALDID according to the ratio of training set: validation set = 4:1 for model training and validation. The training set is used to train the model, and the validation set is used to check whether the model training process converges normally and whether there is an overfitting problem.

**Table 1:** The composition of apple leaf disease identification data set (ALDID).

**Category**	**Healthy**	**Mosaic**	**Rust**	**Glomerella leaf spot**	**Black rot**	**Scab**	**Apple litura moth**	**Apple leaf mites**
Training	8,240	8,240	7,000	8,360	7,000	7,000	8,200	8,320
Validation	2,060	2,060	2,000	2,090	2,000	2,000	2,050	2,080
Total	10,300	10,300	10,000	10,450	10,000	10,000	10,250	10,400

During the training process, a large fluctuation of the feature value range will affect the convergence of the model, which is not conducive to the model learning different feature differences, and the images need normalization. In order to test the stability of the model, 500 images were randomly selected from each type of disease image in the original data set, and a total of 4,000 images were selected from eight different diseases. After scrambling these 4,000 images, five different interference factors, namely, Gaussian noise, salt and pepper noise, 180° rotation, 30% sharpness enhancement, and 30% sharpness reduction were randomly added, and a Model Robustness Test Data set (MRTD) was generated. After the training process is completed, the MRTD is then used to test the model to verify the effect of the model training. The above work laid the foundation for the use of the model.

### CA-ENet Network

The existing CNN methods of increasing network depth, width, and input image resolution can obtain richer and higher fine-grained features, but, there will be serious problems such as gradient disappearance and model degradation. The problem is that only changing a single variable cannot achieve better results. The basic network architecture EfficientNet-B0 (Tan and Quoc, 2019), which uses neural architecture search (NAS) techniques to optimize the above three factors at the same time, balances the three dimensions of depth, width, and resolution, and can be further adjusted by the scaling factor. Therefore, in this study, we use the EfficientNet architecture as the feature extraction network.

**Figure 3:**
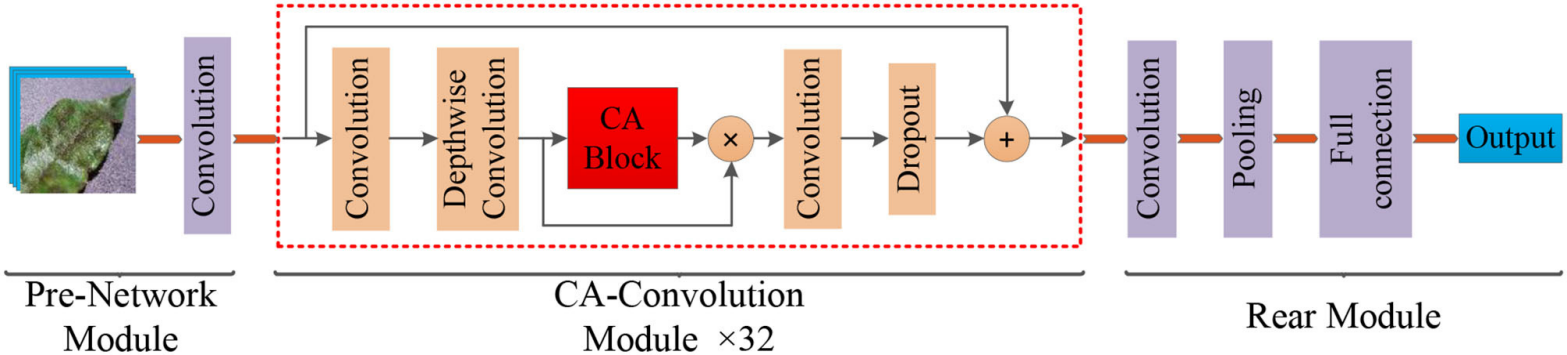
Structure of the Coordination Attention EfficientNet (CA-ENet) for apple disease identification.

Different types of apple leaf diseases have different morphological characteristics with regard to lesions, but there is a high degree of similarity between certain types of diseases, which means apple disease classification can be viewed as a fine-grained image classification problem, and existing models still have difficulty achieving satisfactory results. Therefore, in order to enhance model effectiveness, attention to the lesion area is the key to solving this problem. The widely used channel attention mechanism, SENet (Hu et al., 2018), has a significant effect on improving final performance, but this operation ignored the location information of the features, which is also important for generating spatial selective attention maps. In order to identify these differences, the CA-ENet is proposed to achieve real-time and accurate apple disease identification. The overall structure of the model is shown in Figure 3.

The model mainly included three parts: the pre-network for the Batch Normalization of input images, the backbone network CA-Conv for feature extraction, and the rear part that outputs the recognition result through the fully connected layer. Pre-network uses a layer of 3 × 3 ordinary convolutions with a step of 1 to perform the convolution operation on the input image, the input image resolution is 380 × 380, and the feature map with the depth of the output feature matrix of 48 is obtained. Then, the obtained feature matrix are input into the 32 CA-Conv module embedded with the CA block. Finally, the 3 × 3 ordinary convolutions and pooling are used to further abstract features and then output through a fully connected layer with eight nodes.

During the model optimization process, a NAS technique is used to search for the optimal model structure. The operation process can be abstractly expressed as Equation (1):


(1) $$\begin{array}{l} \text{N}\left(\text{d},\text{w},\text{r}\right)={⊙}_{\text{i}=1,2,\ldots ,\text{s}}{\text{F}}_{\text{i}}^{{\text{L}}_{\text{i}}}\left({\text{X}}_{\left({\text{H}}_{\text{i}},{\text{W}}_{\text{i}},{\text{C}}_{\text{i}}\right)}\right) \end{array}$$

where ⊙ is the multiplication symbol. *F*${}_{i}^{Li}$ means arithmetic operation, it is repeatedly executed *L*_*i*_ times in the operation *F*_*i*_. *X* is the input feature matrix. (*H*_*i*_*, W*_*i*_*, and C*_*i*_) represents the height, width, and output channels of *X*. The NAS process can be optimized by adding the constraints of model accuracy, parameter, and calculation amount with Equations (2) and (5).


(2) $$\begin{array}{l} \max_{\left(\text{d},\text{w},\text{r}\right)}\left[\text{Accuracy}\left(\text{N}\left(\text{d},\text{w},\text{r}\right)\right)\right] \end{array}$$

(3) $$\begin{array}{l} \text{N}\left(\text{d},\text{w},\text{r}\right)={⊙}_{\text{i}=1,2,\ldots ,\text{s}}{\hat{\text{F}}}_{\text{i}}^{\text{d}\cdot {\hat{\text{L}}}_{\text{i}}}\left({\text{X}}_{\left(\text{r}\cdot {\hat{\text{H}}}_{\text{i}},\text{r}\cdot {\hat{\text{W}}}_{\text{i}},\text{r}\cdot {\hat{\text{C}}}_{\text{i}}\right)}\right) \end{array}$$

(4) $$\begin{array}{l} \text{Memory}\left(\text{N}\right)\leq \text{tar\_memory} \end{array}$$

(5) $$\begin{array}{l} \text{FLOPs}\left(\text{N}\right)\leq \text{tar\_flops} \end{array}$$

The *d, w*, and *r* are the sparseness that scales the depth, width, and resolution of the network, respectively, the *tar_memory* and *tar_flops* are the constraints on the number of parameters and calculations. Through the above optimization calculation, the best *d, w*, and *r* values of the EfficientNet-B0 structure can be obtained, and on this basis, the magnification factors *d* and *w* of EfficientNet-B4 are 1.8 and 1.4, respectively, and the input image resolution *r* is 380 × 380 pixels. From the discussed method, the optimal CA-ENet structure parameters can be calculated and are shown in Table 2.

**Table 2:** Details about coordination attention EfficientNet (CA-ENet).

**Stage**	**Operator**	**Resolution**	**Output channels**	**Repeat**	**First-stride**
1	Conv, 3 × 3	380 × 380	48	1	2
2	CA-Conv1, 3 × 3	190 × 190	24	2	1
3	CA-Conv6, 3 × 3	190 × 190	32	4	2
4	CA-Conv6, 5 × 5	95 × 95	56	4	2
5	CA-Conv6, 3 × 3	48 × 48	112	6	2
6	CA-Conv6, 5 × 5	24 × 24	160	6	1
7	CA-Conv6, 5 × 5	24 × 24	272	8	2
8	CA-Conv6, 3 × 3	12 × 12	448	2	1
9	Conv, 3 × 3	12 × 12	1,792	1	1
10	Avg Pooling, 1 × 1	12 × 12	1,792	1	1
11	fc	1 × 1 × 1,792	8	1	1

**Figure 4:**
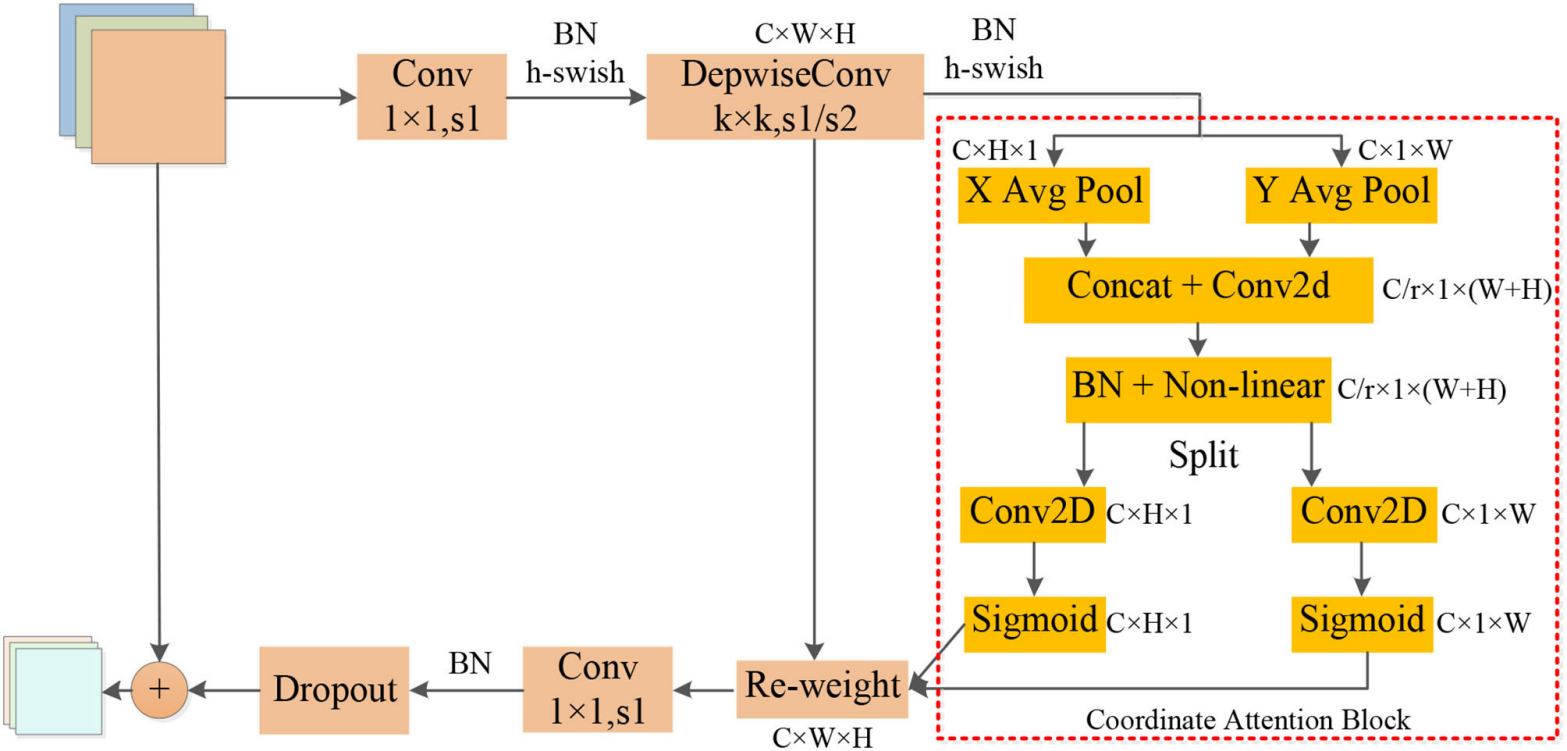
Structure of coordination attention convolutional (CA-Conv).

The operators in Table 2 perform arithmetic operations on the input features. The magnification of each CA-Conv6 in Stage 3–Stage 8 is 6; that is, in the first layer of convolution, the depth of the feature matrix of the input layer is increased to 6 times of the input, and the size of the convolution kernel is 3 × 3 or 5 × 5. The resolution, output channels, and repeat correspond to the resolution of the input layer, the depth of the output feature matrix, and the number of repetitions of the layer structure in the depth direction. The steps given by first-stride are only for the first layer structure of each stage, and the steps of the other layer structures are all 1. The network is composed of seven-stage CA-Conv blocks, and its structure is shown in Figure 4. First, the input feature matrix is sent to CA-Conv through an ordinary 1 × 1 convolution for dimension upgrade. After the h-swish activation function, the feature is extracted through the deep separable convolution with a convolution kernel size of k × k (k = 3 or 5) and a step of 1 or 2. The use of a deep separable convolution structure greatly reduces the number of model parameters, and at the same time, can play an important role in avoiding model overfitting. Then, the obtained feature matrix is divided into two branches, one of which is assigned a weight to each channel by a Coordinate Attention Block (CAB), and another one without any processing is multiplied by the two weights passed through the CAB to obtain the weighted feature matrix. Finally, the dimension is reduced by 1 × 1 convolution and output to the subsequent structure after adding with the input feature matrix.

The global pooling method can compress the global spatial information into the channel descriptor, but this results in a lack of location information. In order to capture the precise location information of the features, in the CAB in Figure 4, the global pooling is decomposed into two one-dimensional feature encoding processes according to Equation (6). Furthermore, two one-dimensional average pooling operations along the horizontal and vertical directions are used to aggregate the input features into two separate direction-aware feature maps. This operation captures both direction-aware and position-sensitive information, thus enabling the model to locate the region of interest more accurately. The generated two separate direction-aware feature maps are concatenated in the depth direction, and the feature channel attention weight is generated through a 1 × 1 convolution compression channel, and the position information is embedded in the channel attention. Then, the Batch Nomalization (BN) operation is applied to the feature matrix and divided into two parts through a non-linear activation function, the feature depth is adjusted to be consistent with the input feature through 1 × 1 convolution, and the position information is saved in the generated attention map. Finally, the weights of the two attention maps are multiplied by the input features to strengthen the feature representation of the attention region and improve the ability of the network to locate the regions of interest accurately.


(6) $$\begin{array}{l} {\text{z}}_{\text{c}}=\frac{1}{\text{H}\times \text{W}}\overset{\text{H}}{\underset{\text{i}=1}{\sum }}\overset{\text{W}}{\underset{\text{i}=1}{\sum }}{\text{x}}_{\text{c}}\left(\text{i},\text{j}\right) \end{array}$$

As the above-mentioned information embedding method can directly obtain the global receptive field and encode the accurate position information, so the transformation operation is performed on it using the 1 × 1 convolution transformation function *F*_1_. As shown in Equation (7), [*z*^*h*^*, z*^*w*^] is the splicing operation along a spatial dimension, δ is the non-linear activation function, and *f* is the intermediate feature map that encodes the spatial information in both horizontal and vertical directions. Then, through two 1 × 1 convolutions, *f* ^*h*^ and *f* ^*w*^ are transformed into tensors with the same number of channels, respectively. As shown in Equations (8) and (9), attention weights can be calculated, and the output of the CA block after the Re-weight is calculated by Equation (10).


(7) $$\begin{array}{l} \text{f}=\delta \left({\text{F}}_{1}\left(\left[{\text{z}}^{\text{h}}{,\text{z}}^{\text{w}}\right]\right)\right) \end{array}$$

(8) $$\begin{array}{l} {\text{g}}^{\text{h}}=\sigma \left({\text{F}}_{\text{h}}\left({\text{f}}^{\text{h}}\right)\right) \end{array}$$

(9) $$\begin{array}{l} {\text{g}}^{\text{w}}=\sigma \left({\text{F}}_{\text{w}}\left({\text{f}}^{\text{w}}\right)\right) \end{array}$$

(10) $$\begin{array}{l} {\text{y}}_{\text{c}}\left(\text{i},\text{j}\right)={\text{x}}_{\text{c}}\left(\text{i},\text{j}\right)\times {\text{g}}_{\text{c}}^{\text{h}}\left(\text{i}\right)\times {\text{g}}_{\text{c}}^{\text{w}}\left(\text{i}\right) \end{array}$$

**Figure 5:**
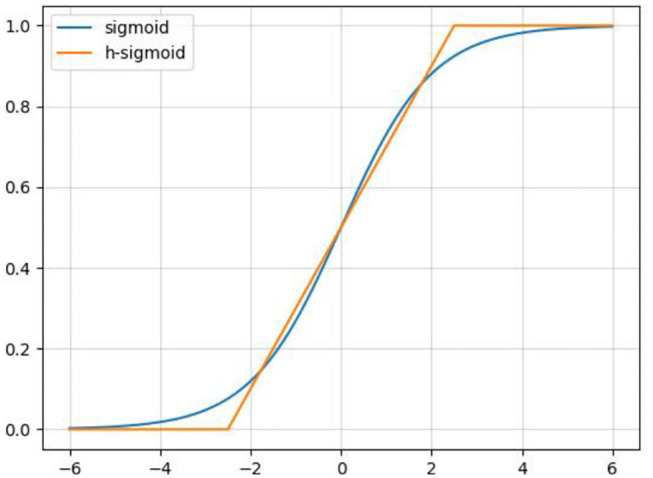
Schematic diagram of the sigmoid and h-sigmoid activation functions.

**Figure 6:**
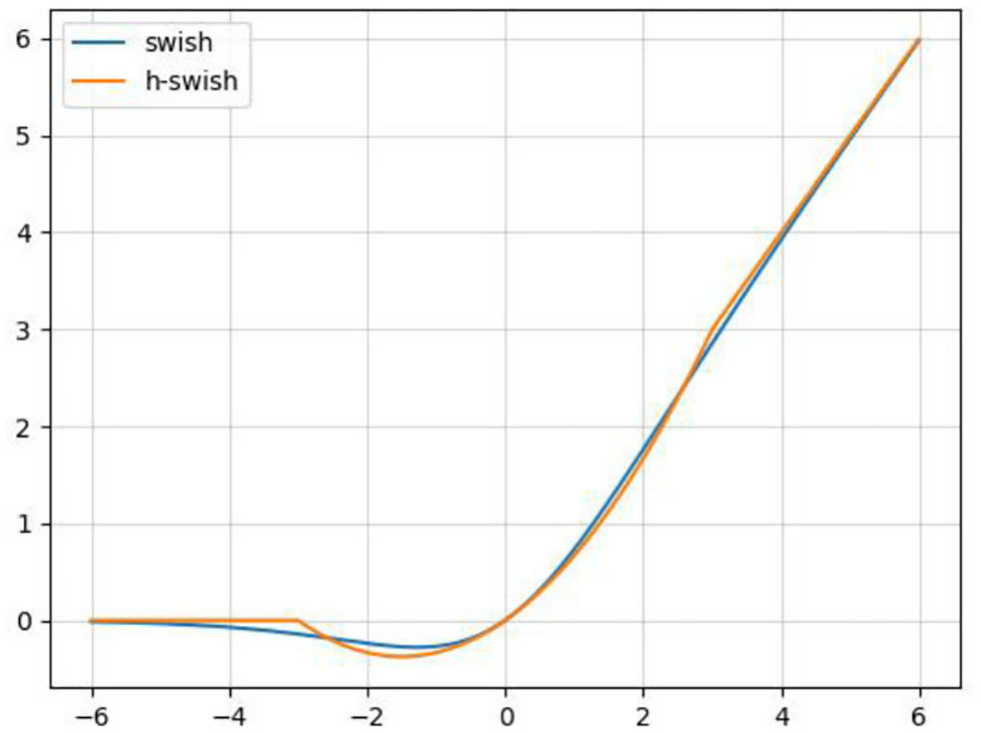
Schematic diagram of the swish and h-swish activation functions.

In order to reduce the amount of calculation and speed up reasoning while ensuring the effect of the activation function, a new activation function, h-swish, is applied into CA-Conv (Howard et al., 2019). The activation functions of sigmoid and h-sigmoid are shown in Equations (11) and (12). It can be seen from Figure 5 that the above two activation functions are relatively close and the calculation process of h-sigmoid is more concise, so h-sigmoid can be used to replace sigmoid in Equations (13) and (14). Figure 6 shows the approximation of the effect of h-swish on the swish activation function. It can be seen that the two curves are basically the same, and the calculation speed of the h-swish is faster.


(11) $$\begin{array}{l} \text{sigmiod }\left(\text{x}\right)=\frac{1}{1+{\text{e}}^{-\text{x}}} \end{array}$$

(12) $$\begin{array}{l} \text{h}-\text{sigmiod }\left(\text{x}\right)=\frac{\text{relu}6\left(\text{x}+3\right)}{6} \end{array}$$

(13) $$\begin{array}{l} \text{swish }\left(\text{x}\right)=\text{x}\cdot \text{sigmoid}\left(\text{x}\right) \end{array}$$

(14) $$\begin{array}{l} \text{h}-\text{swish }\left(\text{x}\right)=\text{x}\cdot \left(\text{h}-\text{sigmoid}\right) \end{array}$$

## Experimental Results and Discussion

### Model Training Details

In order to verify the performance of the proposed method, a proposed network is trained *via* the ALDID. Thus, the proposed method is realized on the Pytorch 1.7.1 deep learning framework, while all experiments were conducted on an Intel® Xeon(R) Gold 5217 CPU@3.00 GHz server equipped with an NVIDIA Tesla V100 (32GB) GPU. The operating system is Ubuntu 18.04.5 LTS 64. In order to accelerate the model convergence while keeping stable training, the initial learning rate is set to .01, and it decays according to the cosine learning rate change curve during the training process, and finally decays to .001. The number of training iterations for all models is 50 epochs.

### Performance of Proposed CA-ENet

In order to evaluate the performance of the proposed method, multiple state-of-the-art methods were applied to the MRTD. In order to ensure that the results are comparable, the same training strategy was used. The test result is visually displayed with a confusion matrix. In order to facilitate the display of labels, the full names of some diseases are abbreviated. In this case, “GLS” in the confusion matrix stands for Glomerella leaf spot, “ALM” stands for Apple litura moth, and “ALMS” stands for Apple leaf mites.

**Figure 7:**
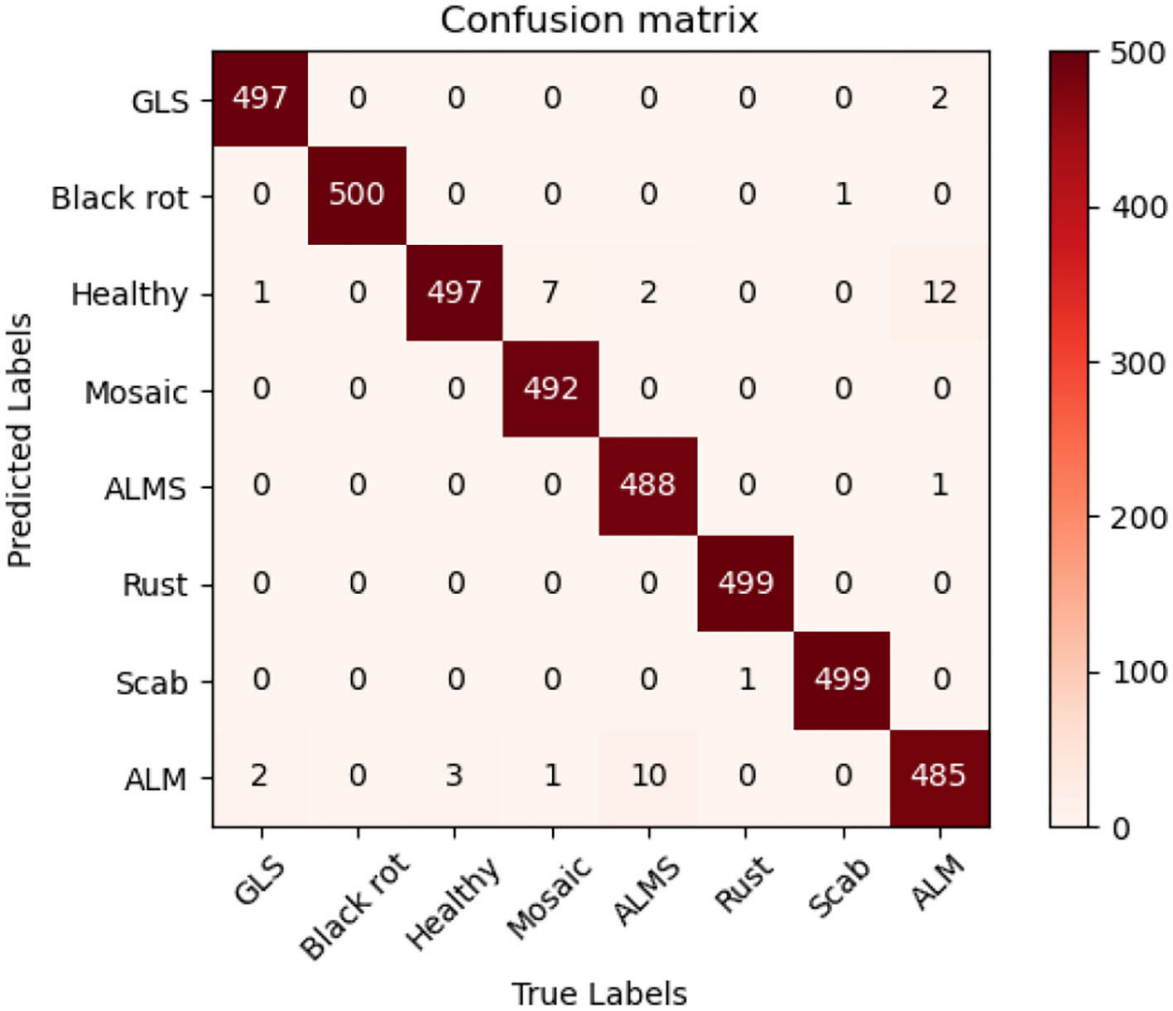
Confusion matrix of the CA-ENet.

Figure 7 can intuitively show the classification performance of the Coordination Attention EfficientNet, with the final accuracy reaching 98.92%. The misclassification mainly occurred between Apple leaf mites and Apple litura moth and between Apple litura moth and Healthy leaves. The main feature of the apple leaf mites is that the damaged leaves show many dense chlorosis gray-white spots. In contrast, after being damaged by apple litura moth, the insect spots formed on the leaves were elliptical and dense, and the leaf surface was wrinkled. The above two kinds of leaf spots have certain similarities in geometric and color characteristics, leading to misjudgment. Furthermore, affected by the complex background, a small number of leaves damaged by apple litura moths were mistakenly identified as healthy leaves. It can be seen that accurate recognition in a complex background has been a great challenge, but the number of misjudgments in this model is still within an acceptable range and can be maintained at a low level. The proposed CA-Conv structure can extract richer fine-grained features of the image and perceive the regions of interest with a higher degree of attention. It can also be seen that the model shows a good recognition effect and has strong robustness to the problem of apple leaf disease recognition.

### Performance Comparison

The performance comparison between CA-ENet and the standard method is shown in Table 3. It can be seen from Table 3 that the proposed model has the best recognition performance on MRTD, with an accuracy of 98.92%. In this study, multiple metrics including accuracy, precision, recall, F1-score, parameter, and calculation are used as evaluation indicators. ResNet-152 takes advantage of the residual structure to make sure it has a strong feature learning ability, so it can reach an accuracy of 93.75%. The Dense Block, the basic structure of DenseNet-264, also has the advantages of enhanced feature propagation and incentive feature reuse, making it achieve a higher accuracy rate with nearly half of the parameters of ResNet-152. Furthermore, the accuracy of ResNeXt-101 reaches 95.67%, which is due to the use of grouped convolution, so it can achieve better results with fewer convolutional layers than ResNet-152. Although this structure can improve the final accuracy, the degree of network fragmentation is very high due to the existence of a large number of parallel branches, which greatly reduces the computation efficiency of the model.

**Table 3:** Performance comparison of the CA-ENet with other classical networks.

	**Accuracy/%**	**Average precision/%**	**Average recall/%**	**Average F1-score**	**Params/M**	**FLOPs/B**
ResNet-152	93.75	94.01	93.75	0.936	60	11.0
DenseNet-264	94.90	95.27	94.90	0.949	34	6.0
ResNeXt-101	95.67	96.05	95.67	0.957	84	32.0
EfficientNet-B4	97.27	97.41	97.27	0.972	19	4.2
CA-ENet	**98.92**	**98.95**	**98.92**	**0.988**	21	4.3

*Bold values indicate the best results under each index*.

EfficientNet uses NAS techniques to simultaneously search and optimize model depth, width, and input image resolution, and rationally expand the model architecture to achieve a high degree of coordination of structural proportions. It has obvious advantages in extracting more robust and reliable feature representations and can reach an overall accuracy of 97.27%. The strong learning ability of the CA module in CA-Conv may cause attention drift and affect model convergence, while the inverted residual structure in CA-Conv can suppress features that are not conducive to classification, ensuring model stability while further improving the recognition performance, and the effectiveness of the attention mechanism is verified.

Traditional CNNs do not distinguish the importance of information when extracting disease features, and there is a large number of convolutions that repeatedly extract low-contribution information, which causes a waste of computation resources. The attention mechanism can automatically extract high-contribution feature components, with only small parameters and calculations increases. The experimental results also show that, in the identification of apple leaf diseases, the proposed CA-ENet model is superior to other models in all evaluation indicators with fewer parameters and can classify apple disease images more accurately.

### Effect of Data Augmentation on Identification Performance for Each Class

A variety of data expansion methods is used in the ALDID to improve the anti-interference ability of the model in complex situations and prevent the problem of overfitting. In order to verify the effect of data augmentation, a set of comparative experiments is designed to evaluate its impact on the final classification performance. Table 4 shows the accuracy, recall, and F1-score performance indicators of the proposed model for each category on the MRTD. The first row of values in each performance index is the performance obtained after training on the original dataset, and the second row of values is the performance obtained after training on the ALDID. It can be seen from Table 4 that the image diversity of the original data set is insufficient, and the average F1-score of the proposed method on the original dataset is 0.969, which is slightly lower than the performance of the model obtained on the ALDID, but it can still accurately classify apple leaf diseases. The results show that the augmented data set is closer to the actual situation, the ability of the model to adapt to complex scenes is enhanced, and the anti-interference ability is improved to a certain extent. The leverage of the deep separable convolution can effectively reduce the number of model parameters and greatly increase training speed.

**Table 4:** Performance of the CA-ENet before and after data augmentation.

**Dataset**	**Metrics**	**Glomerella leaf spot**	**Black rot**	**Healthy**	**Mosaic**	**Apple leaf mites**	**Rust**	**Scab**	**Apple litura moth**
Original	Precision/%	96.9	93.5	99.1	95.0	96.1	100.0	100.0	96.0
		99.6	99.8	95.8	100.0	99.8	100.0	99.8	96.8
	Recall/%	99.0	100.0	89.6	99.4	99.2	99.7	92.8	95.8
ALDID		99.4	100.0	99.4	98.4	97.6	99.8	99.8	97.0
	F1-score	0.979	0.966	0.941	0.972	0.976	0.998	0.963	0.959
		**0.995**	**0.989**	**0.976**	**0.992**	**0.987**	**0.999**	**0.998**	**0.969**

*Bold values indicate the best results under each index*.

### Feature and Network Attention Visualization

**Figure 8:**
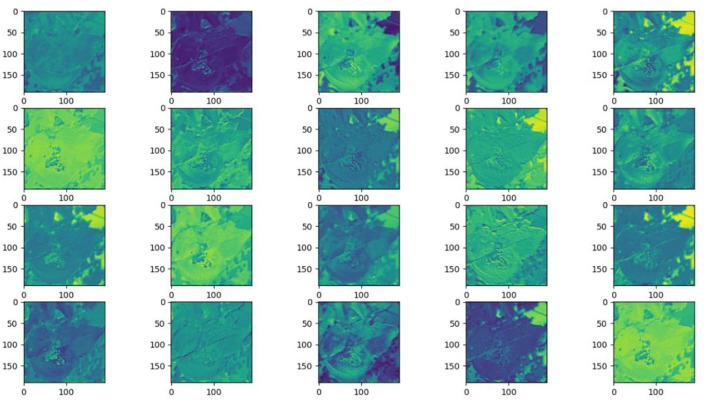
Partial output feature maps of the first CA-Conv.

**Figure 9:**
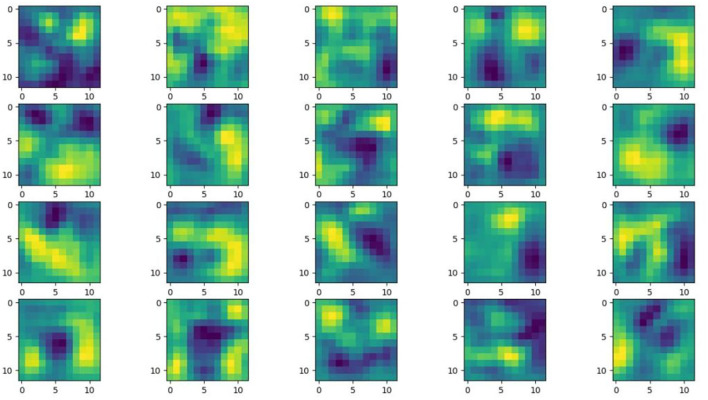
Partial output feature maps of the last CA-Conv.

Understanding and analyzing the hidden layer structure of the model is an important method to comprehensively recognize the proposed network structure. CNNs are usually trained in the form of black-box testing and the evaluations of model performance are limited to the final accuracy and other indicators, which have certain deficiencies. Visualization techniques are the way to explore how CNNs learn features and distinguish categories. So, this section uses the visualization of layer activation and class activation heatmaps to analyze the performance of the proposed model. The visualization of layer activation helps to understand how the continuous convolutional layer performs feature extraction and completes the conversion of input features. Figures 8, 9 show the output features of the first 20 channels of the CA-Conv structure in the first and last layers of the model, respectively. The given example category is apple leaf mites. In the superficial features of the model, it is obvious that the lesion area and the background are separated, and the characteristics of the disease location can be accurately extracted. The model has high efficiency in extracting deep features and only contains a few failed convolutions. The channel output features given here are all valid. Therefore, the stacking of the CA-Conv structure does not affect feature learning ability of the model, and the adopted separable convolution can effectively reduce the feature redundancy and lead to higher efficiency.

Class Activation Mapping (CAM) (Selvaraju et al., 2020) helps to understand which feature components the model relies on to make decisions. Table 5 shows the original image of the class activation and the attention heatmaps of the commonly used models. The sample images of Glomerella leaf spot, black rot, apple litura moth, and rust are randomly selected for testing. Due to the introduction of the attention module CAB, CA-ENet has a stronger ability to focus on the lesion area. Compared with other models, CA-ENet has a good positioning effect and can accurately locate the interest area, whether it is a leaf lesion in a complex or a simple background. In contrast, ResNet-152, DenseNet-264, and ResNeXt-101 have deviations or even errors in their focus positions, which are what affect the robustness and accuracy of a model. The visual test results of the class activation heatmaps of the apple leaf diseases show that the model fully takes the characteristics of the disease spots into account and achieves superior recognition performance on apple leaf diseases.

**Table 5:** Comparison of attention heatmaps of different models.

**Class**	**Original image**	**ResNet-152**	**DenseNet-264**	**ResNeXt-101**	**CA-ENet**
Glomerella leaf spot	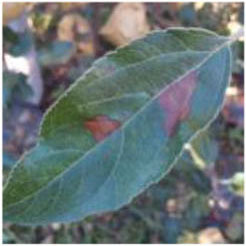	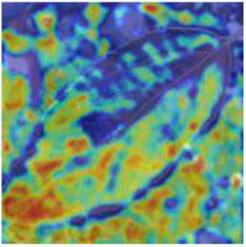	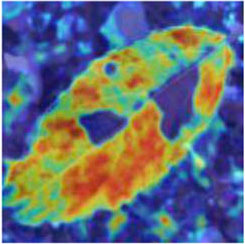	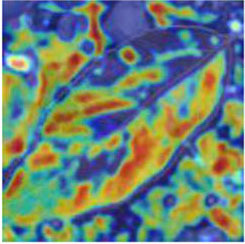	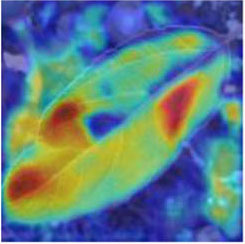
Black rot	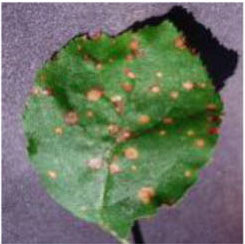	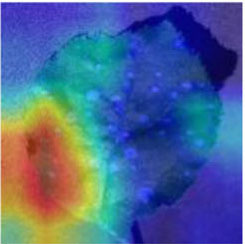	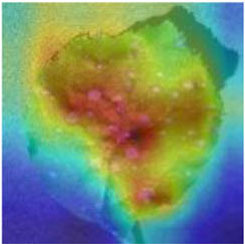	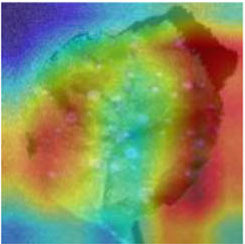	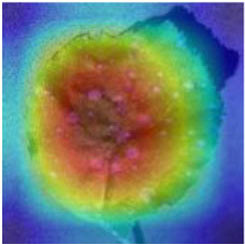
Apple litura moth	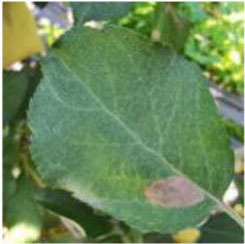	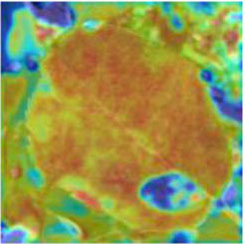	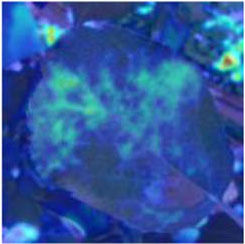	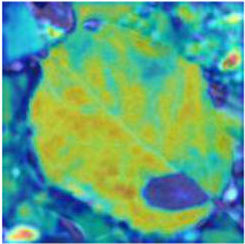	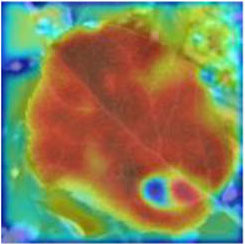
Rust	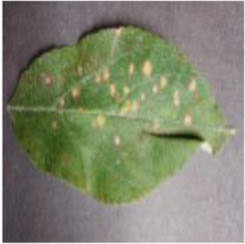	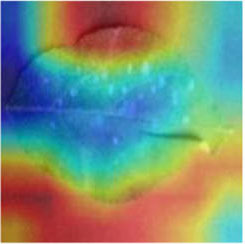	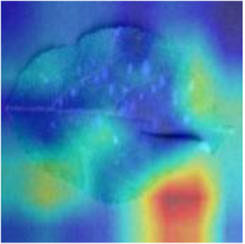	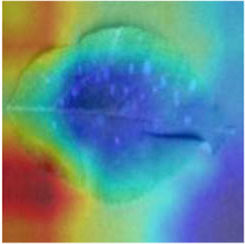	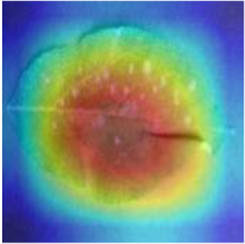

## Conclusion and Future Work

An improved attention-based deep CNN to identify common apple leaf diseases to support the efficient management of orchards is proposed in this study. Due to the complex environment of orchards, in order to be close to the real application scenarios, 5,170 apple leaf images were collected by multiple mobile devices and 3,000 disease images were obtained from a public dataset. Image augmentation techniques are used to generate the ALDID containing 81,700 diseased images. By embedding a CA block into a CA-Conv module, the integration of characteristic channel and location information was realized. A deep separable convolution is also used to reduce the number of parameters, and the h-swish activation function is used to speed up the model convergence. The proposed model is training with ALDID and testing with MRTD and conducts a large number of comparative experiments including various performance evaluation indicators and process visualizations. The experimental results show that the method proposed in this study achieves a recognition accuracy of 98.92%, which is better than that of other existing deep learning methods and achieves competitive performance on apple leaf disease identification tasks, which provides a reference for the application of deep learning methods in crop disease classification. The proposed model has the advantages of a simple structure, fast running speed, good generalization performance, and robustness, and has great potential application value. In the future, a ground mobile inspection platform equipped with cameras will be built to replace manual operations and to realize the rapid diagnosis and early warning of apple diseases.

## Data Availability Statement

The original contributions presented in the study are included in the article/supplementary material, further inquiries can be directed to the corresponding authors.

## Author Contributions

PW designed and performed the experiment, selected the algorithm, analyzed the data, trained the algorithms, and wrote the manuscript. PW, TN, YM, and ZZ collected data. BL monitored the data analysis. DH conceived the study and participated in its design. All authors contributed to the article and approved the submitted version.

## Funding

This research is sponsored by the Key Research and Development Program of Shaanxi (2021NY-138 and 2019ZDLNY07-06-01), by CCF-Baidu Open Fund (NO. 2021PP15002000).

## Conflict of Interest

The authors declare that the research was conducted in the absence of any commercial or financial relationships that could be construed as a potential conflict of interest.

## Publisher's Note

All claims expressed in this article are solely those of the authors and do not necessarily represent those of their affiliated organizations, or those of the publisher, the editors and the reviewers. Any product that may be evaluated in this article, or claim that may be made by its manufacturer, is not guaranteed or endorsed by the publisher.
